# Correlation analysis of *RDM1* gene with immune infiltration and clinical prognosis of hepatocellular carcinoma

**DOI:** 10.1042/BSR20203978

**Published:** 2021-09-17

**Authors:** Chen Qiu, Zuyin Li, Wanyue Cao, Xiaoni Cai, Li Ye, Cheng Zhang, Yuefeng Ma, Xiaoliang Wang, Yulong Yang

**Affiliations:** 1 Center of Gallbladder Disease, Shanghai East Hospital, Institute of Gallstone Disease, School of Medicine, Tongji University, Shanghai 200092, China; 2 Department of General Surgery, Shanghai General Hospital, Shanghai Jiao Tong University School of Medicine, Shanghai 201600, China; 3 Department of General Surgery, Shaoxing Shangyu People’s Hospital, Shaoxing 312300, China; 4 Department of General Surgery, Affiliated Zhongshan Hospital of Dalian University, Dalian University, Dalian 116001, China; 5 Department of General Surgery, Shanghai Pudong Hospital, Fudan University Affiliated Pudong Medical Center, Shanghai 201399, China

**Keywords:** cancer, immune infiltration, iver hepatocellular carcinoma

## Abstract

**Purpose**: Liver hepatocellular carcinoma (LIHC) is one of the most common primary malignant liver tumors worldwide. The RAD52 motif-containing protein 1 (RDM1) has been shown to play a role in mediating DNA damage repair and homologous recombination. The present study was designed to determine the expression of RDM1 and its prognostic value as well as its relationship with immune infiltration in LIHC patients.

**Methods**: Oncomine and Tumor Immunoassay Resource were used to assess the expression of RDM1. PrognoScan and Kaplan–Meier bioinformatics database were used to analyze the impact of clinical influencing factors on prognosis. Finally, the Tumor Immune Assessment Resource (TIMER) and Gene Expression Analysis Interactive Analysis (GEPIA) databases were used to detect the correlation between the expression of RDM1 and expression of marker genes related to immune infiltration. Immunohistochemistry (IHC) method was used to detect the expression level of RDM1 in 90 cases of hepatocellular carcinoma and adjacent normal liver tissues.

**Results**: RDM1 expression was up-regulated in most cancers. The expression of RDM1 was remarkably higher than that of the corresponding normal control genes in LIHC tissues. The increase in RDM1 messenger RNA (mRNA) expression was closely related to the decreases in overall survival (OS) and progression-free survival (PFS). Additionally, the increase in RDM1 mRNA expression was closely related to the infiltration levels of macrophages, CD8^+^ T cells and B cells and was positively correlated with a variety of immune markers in LIHC.

**Conclusion**: The findings of the present study demonstrate that RDM1 is a potentially valuable prognostic biomarker that can help determine the progression of cancer and is associated with immune cell infiltration in LIHC.

## Introduction

Liver hepatocellular carcinoma (LIHC) is the second leading cause of cancer-related deaths in the world [1]. This global problem can cause more than 700000 deaths every year [2]. Due to the late diagnosis of LIHC, rapid tumor growth and early postoperative recurrence, clinical treatment methods are limited. Treatment methods for hepatocellular carcinoma include partial hepatectomy, liver chemotherapy, liver radiofrequency ablation (RFA), transarterial hepatic chemoembolization (TACE), liver transplantation and molecular targeted therapy. Patients usually receive a combination of these treatments; however, these treatments are ineffective for patients with advanced LIHC [3]. Furthermore, the high rate of disease recurrence in most LIHC patients also leads to poor prognosis [4,5]. Therefore, we urgently need to explore the mechanisms that lead to LIHC and identify biomarkers that can be used for early detection as well as new treatments to block the occurrence and development of LIHC.

In recent years, a large number of studies have shown that immune-related mechanisms play important roles in the development of LIHC, and immunotherapy is considered a valuable direction in the treatment of liver cancer [6–8]. Immunotherapy includes immune cells, antibodies and checkpoint inhibitors. Many studies have shown that increases in infiltrating tumor-associated macrophages (TAMs) and regulatory T cells (Tregs) play indispensable roles in tumor invasion and metastasis, resepectively [9,10]. Meanwhile, checkpoint inhibitors have shown great promise in immunotherapy.

Inhibitors of the programmed cell death protein-1 (PD-1) and programmed death receptor ligand-1 (PD-L1) axis have received increasing attention in clinical studies for the treatment of LIHC. Moreover, serum PD-L1, as a biomarker, can help identify early recurrence and detect treatment effects [11–13]. Therefore, it is necessary to better clarify the immunophenotype of LIHC and better understand the relevant mechanisms of immune cells in the occurrence and development of cancer to identify new immunotherapeutic targets in LIHC.

The gene *RDM1*, which encodes the RAD52 motif protein, is located in region q11.2 of chromosome 17 [14]. RAD52 motif-containing protein 1 (RDM1) contains an RD motif that functionally resembles the N-terminal region of RAD52, a regulator of DNA damage repair [15,16]. Relevant studies have confirmed that RDM1 is involved in resistance to cisplatin, a chemotherapeutic drug. Additionally, RDM1 has the ability to repair damaged DNA through homologous recombination [14,15,17]. Although there are few reports on the developmental role of RDM1 in tumors, studies have highlighted that RDM1 is up-regulated in papillary thyroid cancer, non-small cell lung cancer, ovarian cancer and breast cancer. Knockdown of RDM1 can reduce the proliferation of tumor cells, increase cell apoptosis and induce cell cycle arrest [18–21]. However, the role of RDM1 in LIHC remains largely unexplored.

In the present study, the Oncomine and the Tumor Immune Assessment Resource (TIMER) databases were used to verify the expression level of RDM1 messenger RNA (mRNA) in different tumor tissues compared with normal control tissues. We comprehensively evaluated the correlation between RDM1 expression and patient prognosis as well as the expression of tumor infiltrating immune cells in different types of tumor tissues, including liver cancer, through the use of PrognoScan, Kaplan–Meier plotter, Gene Expression Analysis Interactive Analysis (GEPIA) and TIMER. We combined prognostic analysis and immune infiltration to comprehensively evaluate and judge the role of RDM1 in different cancers, with a focus on hepatocellular carcinoma. In addition, we collected tissues from 90 HCC patients for immunohistochemical staining to further verify the expression of RDM1 protein and its correlation with prognosis and overall survival (OS). The results showed that high expression of RDM1 was a poor prognostic factor for LIHC patients. The present study provides a possible mechanism for the expression of RDM1 in regulating tumor immunity by regulating the infiltration of LIHC immune cells.

## Materials and methods

### Analysis of the expression level of RDM1 in tumor tissues and normal control tissues

Oncomine is a large-scale tumor gene chip database that collects, standardizes and analyzes gene expression profiles from tumor samples. Oncomine integrates RNA and DNA-Seq data from GEO, The Cancer Genome Atlas (TCGA) and the published literature and can also be used to analyze differences in gene expression, predict coexpressed genes etc. (https://www.oncomine.org/resource/login.html) [22]. TIMER analyzes the infiltration of immune cells in tumor tissues from high-throughput sequencing (RNA-Seq expression profiles) data.

TIMER provides several analysis modules, including of gene expression, clinical results, somatic mutations and changes in somatic copy number (https://cistrome.shinyapps.io/timer/) [23]. These two databases were selected to evaluate the RDM1 expression differences in specific tumor types and normal controls.

### Prognostic analysis of RDM1 expression in pan-cancer patients

The PrognoScan database is mainly used to search for relationships between gene expression and patient prognosis in a large number of publicly available cancer chip datasets (http://www.abren.net/PrognoScan/) [24]. These two databases were selected to evaluate the RDM1 expression differences in specific tumor types and normal controls.

Kaplan–Meier plotter is a public database containing mRNA expression profile chips for five types of cancer (breast cancer, ovarian cancer, lung cancer, stomach cancer and liver cancer) [25–29], in which information about gene expression and disease prognosis can be obtained (http://kmplot.com/analysis/). The purposes of this database are to explore the relationship between RDM1 gene expression and survival in the above five tumor types and to analyze the effect of RDM1 gene expression on the prognosis and survival of liver cancer patients when considering different clinicopathological factors. The GEPIA database integrates TCGA data and GTEx normal tissue data, and these data are used to analyze tumor/normal differential expression profiles, expression distribution, pathological staging, survival analysis results, similarity between genes, gene expression correlation etc. (http://gepia.cancer-pku.cn/) [30]. The GEPIA database was used to verify the relationship between RDM1 expression and prognosis across cancer types.

### Analysis of the association between the RDM1 expression level and immune cell infiltrate

TIMER data are based on the TCGA database, which can be used to evaluate the degree of immune infiltration between different cancers by systematically analyzing the expression levels of different immune markers in cancer. Immune cells are an important part of the tumor microenvironment, and tumor infiltrating immune cells are independent predictors of the prognosis of cancer patients. The immune cell infiltrate is related to the clinical characteristics and prognosis of patients [31,32]. The correlations between the RDM1 expression level and B cells, CD8^+^ T cells, CD4^+^ T cells, macrophages, neutrophils and DCs in pan-carcinoma were analyzed. Meanwhile, the GEPIA and TIMER databases were used to jointly verify the correlation between RDM1 expression and liver cancer immune molecular markers; cholangiocarcinoma (CHOL) was used as a reference.

### Immunohistochemical staining

Immunohistochemistry (IHC) used Real™ Envision™ Detection System, Peroxidase/DAB+, Rabbit/Mouse (Dako #5007) detection system. In brief, TMA sections were deparaffinized and rehydrated. After antigen repair, endogenous enzyme blocking, 10% goat serum blocking, antigen–antibody reaction and chromogenic reaction, RDM1 antibody concentration was selected as dilution ratio of 1:250 for staining analysis. The sections were dewatered and dried with gradient alcohol, and the tablets were sealed with environmental protection sealing agent and examined under microscope. Thermo Shandon Finesse 325 was used as paraffin slicer and Olympus Corporation CX31 was used for microscopy. The immunohistochemical staining images were analyzed using CaseViewer integrator system (for Windows 10, version 2.4). In the present study, we scored nucleus staining of epithelial cells by a region of defining tool. An overall immunostaining scores were calculated by multiplying the staining intensity (ranging from 0 to 3) by the percentage of positive cells (ranging from 0 to 4), a total range 0–12. The overall immunohistochemical score was calculated by multiplying the percentage of positive cells by the intensity. IHC score of >6 was regarded as RDM1 high-expression, and the score of 6 was regarded as RDM1 low-expression.

### Statistical analysis

Oncomine, TIMER, PrognoScan, GEPIA and Kaplan–Meier plotter were used to generate corresponding survival maps and immune infiltration maps. The generated results are displayed as the HR and *P*-values or the Cox *P*-value from a log-rank test. Using Spearman’s correlation analysis, the degree of correlation between variables was determined. The absolute value of the correlation was judged as follows: 0.00–0.19, very weak; 0.20–0.30, medium and 0.30–0.50, strong. Values were considered statistically significance when ***P<0.001, **P<0.01, and *P<0.05.

## Results

### Level of RDM1 mRNA in LIHC and other cancers

**Figure 1:**
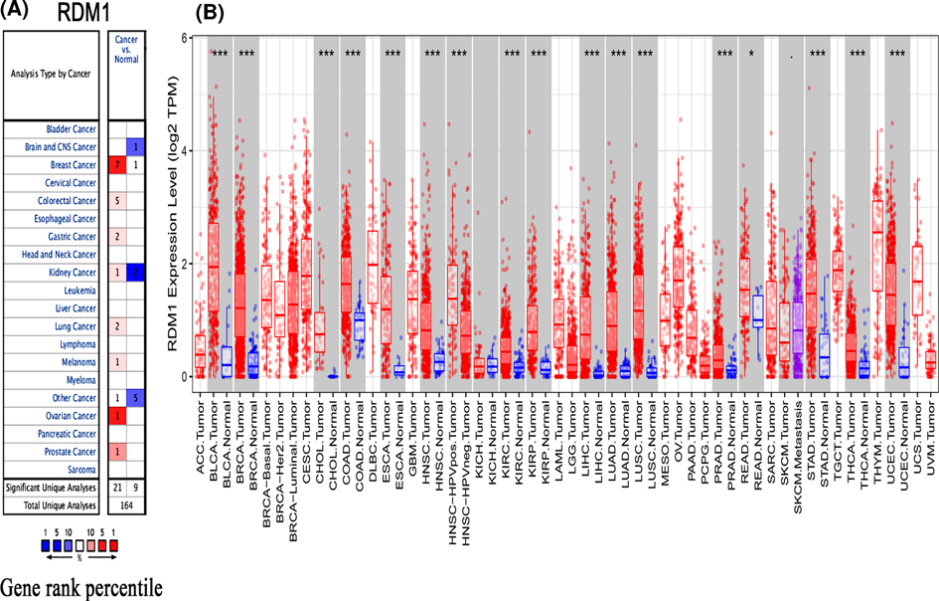
The expression level of RDM1 in different types of human cancers (**A**) High or low expression of RDM1 in different human normal and cancer tissues from Oncomine database. (**B**) The expression level of RDM1 in different types of normal and tumor tissues from TIMER database. *P<0.05, ***P<0.001.

By comparing *RDM1* gene expression between different cancer types and normal tissues using the Oncomine database, we showed that RDM1 is up-regulated in colon cancer, breast cancer, gastric cancer, lung cancer, melanoma, ovarian cancer and prostate cancer. In addition, compared with normal controls, RDM1 is down-regulated in brain cancer and other CNS cancers (Figure 1A and Supplementary Table S1). Then, the TIMER database was used to assess the RDM1 expression differences in different tumor types. The analysis showed that the RDM1 expression levels were significantly higher in the following tumor types than in normal control tissues (*P*<0.001): bladder urothelial carcinoma (BLCA), breast invasive carcinoma (BRCA), CHOL, colon adenocarcinoma (COAD), esophageal cancer (ESCA), head and neck cancer (HNSC), clear renal cell carcinoma (KIRC), kidney renal papillary cell carcinoma (KIRP), hepatocellular carcinoma (LIHC), lung adenocarcinoma (LUAD), squamous cell carcinoma of the lung (LUSC), prostate adenocarcinoma (PRAD), gastric adenocarcinoma (STAD), thyroid carcinoma (THCA) and endometrial carcinoma (UCEC) (Figure 1B).

### Prognostic significance of RDM1 expression across cancers

**Figure 2:**
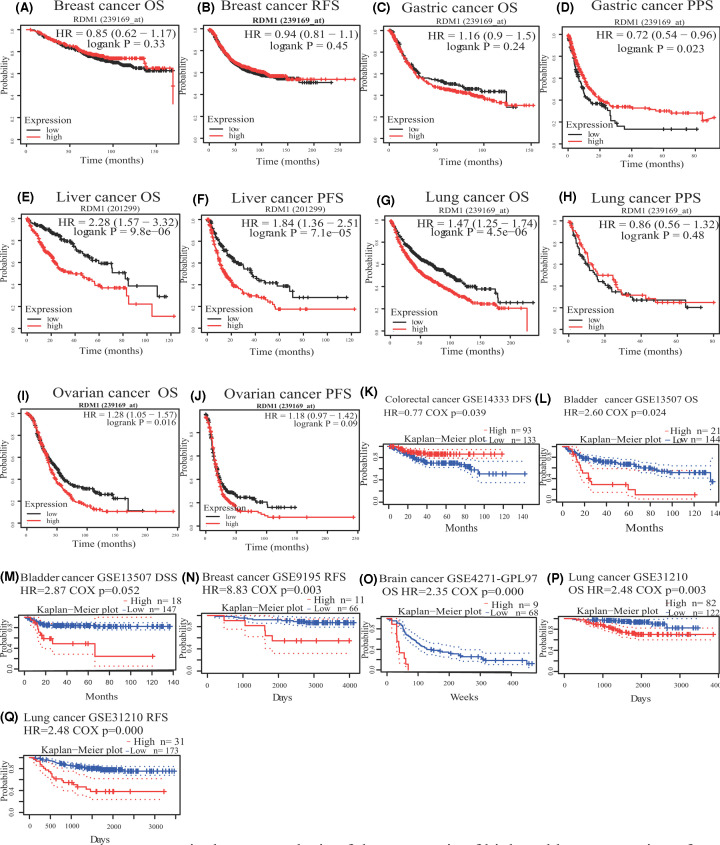
Effects of RDM1 expression on survival Kaplan–Meier survival curve analysis of the prognosis of high and low expression of RDM1 in different cancer types in PrognoScan (**K–Q**) and Kaplan–Meier plotter database (**A–J**).

Subsequently, to fully assess the relationship between RDM1 expression and the prognosis of various cancers, the Kaplan–Meier plotter and PrognoScan databases were used (Supplementary Figures S1–S4). It can be seen that the expression of RDM1 in the Kaplan–Meier plotter database was significantly related to the poor prognosis of LIHC (OS, HR = 2.28, 95% CI = 1.57–3.32, log-rank *P*=9.8e-06; progression-free survival (PFS), HR = 1.84, 95% CI = 1.36–2.51, log-rank *P*=7.1e-05) (Figure 2E,F). Additionally, compared with the correlation of RDM1 expression of breast, stomach, lung or ovarian cancer, RDM1 expression and hepatocellular carcinoma have a higher prognostic HR value, and their correlation is more significant (Figure 2A–D,G-J). The correlation between RDM1 expression and the prognosis of patients with different types of cancer was calculated using the PrognoScan database. It was found that RDM1 expression is related to ovarian cancer, bladder cancer, breast cancer, brain cancer and lung cancer (Figure 2K–Q). At the same time, to expand the sample size, the relationship between RDM1 expression and pan-cancer prognosis was analyzed using the TCGA and GTEx datasets from the GEPIA database. These results showed that RDM1 had a significant effect on prognosis in patients with adrenocortical carcinoma (ACC), KIRP, brain lower grade glioma (LGG) and LIHC. These results clearly indicated that the poor prognosis of a variety of tumors and RDM1 expression are related.

To further explore the effects of high RDM1 expression and different clinicopathological factors on the prognosis of LIHC patients, we studied the correlation between RDM1 expression and different clinical characteristics in LIHC patients. Kaplan–Meier plotter was used for statistical analysis, and the results are shown in Table 1. It was found that the expression of RDM1 was significantly correlated with gender, stage, grade, American Joint Committee on Cancer TNM system-T tumor size (AJCC_T), vascular invasion, alcohol consumption and hepatitis virus status.

**Table 1:** The effect of different clinicopathological factors on RDM1 mRNA expression and prognosis in LIHC was determined by Kaplan–Meier plotter

Clinicopathological factors	OS		*n*=364	PFS		*n*=366
	*n*	Hazard ratio	*P*-value	N	Hazard ratio	*P*-value
Gender						
Male	246	2.63 (1.58–4.37)	1.10E-04	246	1.92 (1.31–2.8)	6.50E-04
Female	118	2.27 (1.28–4)	3.80E-03	120	1.84 (1.08–3.12)	2.30E-02
Stage						
1	170	2.06 (1.1–3.85)	2.10E-02	170	1.65 (1–2.72)	4.80E-02
2	83	2.25 (0.98–5.18)	5.10E-02	84	3.66 (1.44–9.29)	3.50E-03
1+2	253	2.11 (1.26–3.51)	3.60E-03	254	1.75 (1.2–2.55)	3.30E-03
3	83	2.76 (1.27–5. 98)	7.40E-03	83	1.84 (0.96–3.52)	6.20E-02
2+3	166	2.23 (1.34–3.69)	1.50E-03	167	2.23 (1.33–3.72)	1.70E-03
4	4	-	-	5	-	-
3+4	87	2.68 (1.28–5.58)	6.30E-03	88	1.8 (0.96–3.36)	6.20E-02
Grade						
1	55	3.36 (1.26–8.95)	1.10E-02	55	2.17 (0.98–4.8)	5.00E-02
2	174	1.96 (1.15–3.34)	1.20E-02	175	2.29 (1.43–3.67)	3.70E-04
3	118	3.44 (1.59–7.42)	8.10E-04	119	1.67 (0.99–2.82)	5.30E-02
4	12	-	-	12	-	-
AJCC_T						
1	180	2.05 (1.13–3.73)	1.60E-02	180	1.7 (1.05–2.76)	3.00E-02
2	90	2.16 (0.98–4.74)	5.00E-02	92	3.75 (1.6–8.8)	1.10E-03
3	78	2.31 (1.17–4.57)	1.30E-02	78	1.69 (0.89–3.21)	1.00E-01
Vascular invasion						
None	203	1.77 (1.05–2.99)	3.00E-02	204	1.6 (1.03–2.51)	3.70E-02
Micro	90	2.15 (0.94–4.92)	6.30E-02	91	2.8 (1.25–6.26)	8.90E-03
Macro	16	-	-	16	-	-
Alcohol consumption						
Yes	115	2.52 (1.26–5.03)	6.70E-03	115	3.17 (1.63–6.16)	3.50E-04
None	202	2.3 (1.4–3.77)	7.10E-04	204	1.59 (1.04–2.43)	3.20E-02
Hepatitis virus						
Yes	150	1.71 (0.86–3.42)	1.20E-01	152	1.35 (0.82–2.19)	2.30E-01
None	167	3.25 (1.97–5.35)	1.20E-06	167	2.91 (1.8–4.7)	5.90E-06

Particularly, high RDM1 mRNA expression correlated with poor OS and PFS in LIHC patients with stage 1+2, stage 2+3 and stage 3+4 cancer. Additionally, as the stage increased, the hazard ratio increased and the *P*-value became significant. These findings correspond to patients with more tumor foci having increased vascular invasion and an increased risk of extrahepatic metastasis. We also found that RDM1 expression was related to each grade but had a higher hazard ratio in LIHC patients with grade 3, which was closely related to prognosis. It can also be observed in Table 1, LIHC patients with vascular invasion had a higher hazard ratio than those without vascular invasion. In patients with concurrent hepatitis virus, the *P*-value was more statistically significant, and the hazard risk was higher than in patients without hepatitis virus. These findings could be related to the hepatitis virus interfering with the expression of the RDM1 protein and affecting the prognosis of patients with hepatocellular carcinoma. These results suggest that the expression of RDM1 in LIHC patients has prognostic significance based on their clinical characteristics.

### RDM1 expression is correlated with immune cell infiltration in LIHC

**Figure 3:**
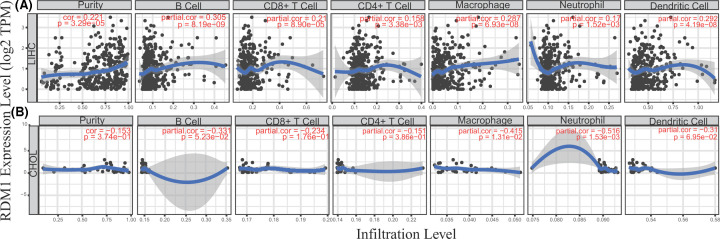
RDM1 expression is correlated with the level of immune infiltration in LIHC and CHOL (**A**) RDM1 expression is correlated with the level of immune infiltration in LIHC. (**B**) RDM1 expression is correlated with the level of immune infiltration in CHOL. *P*<0.05 is considered as significant.

Immune cells are an important part of the tumor microenvironment and play an indispensable role in tumorigenesis and development. Understanding the interaction between immune cells and tumor cells will help develop new tumor treatment strategies [33,34]. In the study of different cancers, many scholars have noted that tumor infiltrating immune cells affect the prognosis of patient survival [35–37]. To explore the relationship between RDM1 expression and immune cell infiltration in 39 cancer types, the TIMER database was used (Figure 3 and Supplementary Figures S5–S9). The data showed that RDM1 expression was significantly correlated with tumor purity in 18 cancer types.

RDM1 expression was also correlated with the degree of immune infiltration of B cells, CD8^+^ T cells, CD4^+^ T cells, macrophages, neutrophils and DCs in 16, 9, 12, 17, 12 and 14 cancer types, respectively. Nevertheless, in CHOL, the relationship between RDM1 and tumor purity was not observed, nor was the expression level of RDM1 correlated with the infiltration of immune cells. In addition, RDM1 expression and LIHC tumor purity (r = 0.221, *P*=3.29e-05) and the infiltration of B cells (r = 0.30, *P*=8.19e-09), CD8^+^ T cells (r = 0.21, *P*=8.90e-05), macrophages (r = 0.29, *P*=6.93e-08) and DCs (r = 0.29, *P*=4.19e-08) showed a significant positive correlation. This correlation was strongest in macrophages and B cells (Figure 3A). These data show that RDM1 plays a key role in regulating the infiltration of LIHC immune cells, especially macrophages.

### Correlation analysis between the RDM1 expression level and immune markers

To further elucidate the potential relationship between RDM1 and infiltrating immune cells, we tested the correlation between RDM1 expression and multiple immune cell markers in the TIMER and GEPIA databases with CHOL as the control group. These immune markers were used to characterize the following types of immune cells: CD8^+^ T cells, T cells (general), B cells, monocytes, TAMs, M1/M2 macrophages, neutrophils, natural killer cells (NK), DCs and other T-cell subsets in LIHC and CHOL.

Considering that tumor purity was an important confounding factor in evaluating the correlation between gene expression and clinicopathological characteristics, tumor purity should be considered and corrected [38,39]. The TIMER database showed that the expression level of RDM1 was closely related to most of the immune markers in various immune cells in LIHC (Table 2), whereas in CHOL, the correlation between the RDM1 expression level and most of the immune markers was not significant (Table 2).

**Table 2:** Correlation analysis between RDM1 and relate genes and markers of immune cells in TIMER

Description	Gene markers	LIHC				CHOL			
		None		Purity		None		Purity	
		Cor	*P*	Cor	*P*	Cor	*P*	Cor	*P*
CD8^+^ T cell	CD8A	0.07	1.55E-01	0.19	**	−0.07	6.89E-01	−0.15	3.75E-01
	CD8B	0.10	5.96E-02	0.21	***	−0.03	8.76E-01	−0.09	6.24E-01
T cell (general)	CD3D	0.18	**	0.31	***	−0.28	9.44E-02	−0.43	*
	CD3E	0.06	2.77E-01	0.21	***	−0.15	3.78E-01	−0.30	7.86E-02
	CD2	0.08	1.44E-01	0.22	***	−0.14	4.10E-01	−0.28	1.07E-01
B cell	CD19	0.18	**	0.27	***	−0.11	5.22E-01	−0.22	2.04E-01
	CD79A	0.04	5.01E-01	0.15	*	−0.14	4.20E-01	−0.24	1.58E-01
Monocyte	CD86	0.15	*	0.30	***	−0.32	6.06E-02	−0.48	*
	CD115 (CSF1R)	0.04	4.82E-01	0.18	**	−0.18	2.87E-01	−0.27	1.17E-01
TAM	CCL2	−0.03	5.33E-01	0.08	1.42E-01	−0.35	*	−0.40	*
	CD68	0.10	5.33E-02	0.19	**	−0.12	4.69E-01	−0.18	3.10E-01
	IL10	0.10	*	0.22	***	0.12	4.70E-01	0.03	8.66E-01
M1 macrophage	INOS (NOS2)	−0.02	7.68E-01	−0.01	9.06E-01	0.08	6.54E-01	0.08	6.62E-01
	IRF5	0.33	***	0.32	***	0.05	7.91E-01	0.01	9.58E-01
	COX2 (PTGS2)	−0.07	1.76E-01	0.06	3.05E-01	0.08	6.43E-01	0.03	8.86E-01
M2 macrophage	CD163	0.00	9.41E-01	0.12	*	−0.26	1.30E-01	−0.39	*
	VSIG4	0.05	3.75E-01	0.17	*	−0.23	1.82E-01	−0.33	5.06E-02
	MS4A4A	0.02	6.89E-01	0.15	*	−0.24	1.56E-01	−0.43	*
Neutrophil	CD66b (CEACAM8)	0.02	7.79E-01	0.05	3.98E-01	0.14	4.18E-01	0.14	4.32E-01
	CD11b (ITGAM)	0.24	***	0.34	***	−0.14	4.23E-01	−0.18	2.93E-01
	CCR7	−0.05	3.01E-01	0.08	1.20E-01	−0.12	4.94E-01	−0.25	1.41E-01
Natural killer cell	KIR2DL1	−0.09	8.63E-02	−0.11	*	0.05	7.86E-01	0.03	8.86E-01
	KIR2DL3	0.07	1.73E-01	0.12	*	−0.05	7.64E-01	−0.07	6.85E-01
	KIR2DL4	0.11	*	0.14	*	0.07	6.73E-01	0.04	8.19E-01
	KIR3DL1	−0.01	7.91E-01	0.01	8.94E-01	0.04	8.33E-01	0.01	9.33E-01
	KIR3DL2	0.00	9.41E-01	0.05	3.33E-01	−0.38	*	−0.39	*
	KIR3DL3	−0.02	6.79E-01	−0.01	8.37E-01	0.20	2.44E-01	0.18	2.95E-01
	KIR2DS4	−0.01	9.06E-01	−0.03	6.21E-01	0.05	7.67E-01	0.03	8.69E-01
Description	HLA-DPB1	0.04	3.94E-01	0.17	*	−0.31	6.86E-02	−0.43	*
	HLA-DQB1	0.08	1.44E-01	0.20	**	−0.14	4.21E-01	−0.19	2.68E-01
	HLA-DRA	0.04	3.96E-01	0.16	*	−0.23	1.84E-01	−0.35	*
	HLA-DPA1	0.02	7.14E-01	0.15	*	−0.20	2.40E-01	−0.32	6.37E-02
	BCDA-1 (CD1C)	−0.05	3.58E-01	0.04	4.06E-01	−0.19	2.72E-01	−0.29	9.08E-02
	BDCA-4 (NRP1)	−0.02	7.18E-01	−0.01	9.18E-01	−0.33	5.07E-02	−0.41	*
	CD11C (ITGAX)	0.15	*	0.28	***	−0.18	2.98E-01	−0.30	7.99E-02
Th1	T-bet (TBX21)	−0.04	4.37E-01	0.06	2.58E-01	−0.07	6.69E-01	−0.21	2.28E-01
	STAT4	0.16	*	0.23	***	0.10	5.63E-01	0.06	7.49E-01
	STAT1	0.20	***	0.25	***	−0.10	5.56E-01	−0.13	4.55E-01
	IFN-γ (IFNG)	0.18	**	0.27	***	0.02	9.12E-01	−0.06	7.32E-01
	TNF-ax (TNF)	0.11	*	0.24	***	−0.32	6.10E-02	−0.35	*
Th2	GATA3	0.02	6.70E-01	0.15	*	−0.15	3.84E-01	−0.29	9.68E-02
	STAT6	−0.02	6.98E-01	−0.04	4.60E-01	−0.19	2.63E-01	−0.19	2.79E-01
	STAT5A	0.16	*	0.21	**	−0.29	8.52E-02	−0.33	5.28E-02
	IL13	0.06	2.40E-01	0.08	1.63E-01	0.25	1.48E-01	0.22	2.10E-01
Tfh	BCL6	0.04	4.18E-01	0.03	6.12E-01	−0.05	7.57E-01	−0.07	7.02E-01
	IL21	0.09	9.85E-02	0.13	*	−0.09	6.04E-01	−0.14	4.26E-01
Th17	STAT3	0.00	9.90E-01	0.03	5.25E-01	−0.32	6.08E-02	−0.32	5.96E-02
	IL17A	0.00	9.88E-01	0.02	6.88E-01	0.22	1.96E-01	0.19	2.70E-01
Treg	FOXP3	0.09	7.08E-02	0.18	**	−0.05	7.67E-01	−0.16	3.71E-01
	CCR8	0.15	*	0.23	***	−0.05	7.86E-01	−0.12	4.91E-01
	STAT5B	0.19	**	0.16	*	−0.09	5.99E-01	−0.11	5.34E-01
	TGFB (TGFB1)	0.06	2.52E-01	0.15	*	−0.30	7.67E-02	−0.36	*
T-cell exhaustion	PD-1 (PDCD1)	0.16	*	0.25	***	−0.13	4.34E-01	−0.19	2.87E-01
	CTLA4	0.21	***	0.33	***	−0.11	5.05E-01	−0.18	3.11E-01
	LAG3	0.24	***	0.31	***	0.05	7.76E-01	−0.01	9.60E-01
	TIM-3 (HAVCR2)	0.16	*	0.31	***	−0.24	1.60E-01	−0.36	*
	GZMB	0.01	8.97E-01	0.08	1.36E-01	−0.07	6.76E-01	−0.16	3.68E-01

Abbreviations: Cor, R value of Spearman’s correlation; Purity, correlation adjusted for tumor purity; Tfh, follicular helper T cell; Th, T helper cell. *P<0.05, **P<0.01, ***P<0.001.

**Figure 4:**
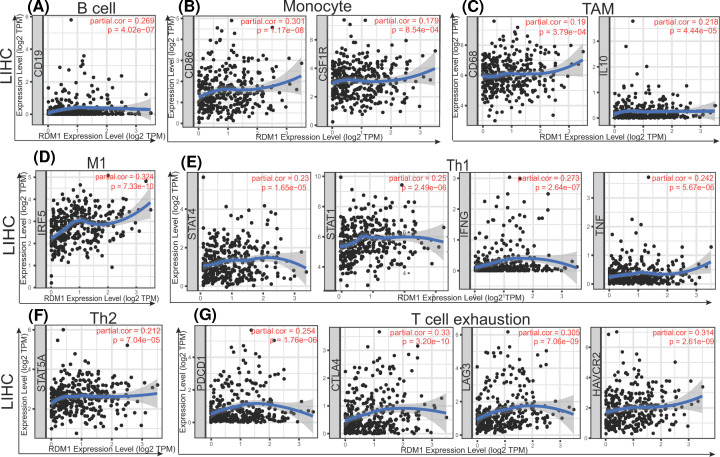
Correlation analysis between RDM1 expression and the expression of immunological marker in LIHC (**A**–**G**) in the TIMER database (**A–G**) Scatterplots of correlations between RDM1 expression and gene markers of B cell (A), monocyte (B), TAM (C), M1 macrophage (D), Th1 (E), Th2 (F) and T-cell exhaustion (G) in LIHC.

Specifically, the expression of RDM1 was related to CD8A, CD8B, CD3D, CD3E, CD2, CD19, CD86, CD115, CD68, IL10IRF5, CD11C, CD11b, HLA-DQB1, STAT4, STAT1, IFN-, TNF-ax, GATA3, STAT5A, FOXP3, CCR8, STAT5B, TGFB, PD-1, CTLA4, LAG3 and TIM-3 according to the analysis of CD8^+^ T cells, general T cells, B cells, monocytes, TAMs, M1 macrophages, M2 macrophages, DCs, Th1 cells, Th2 cells, Tregs and exhausted T cells. We selected immune markers with a strong correlation and displayed them in a scatter diagram with the TIMER database (Figure 4).

Then, the GEPIA database was used to verify these highly relevant immune markers, with CHOL as a reference (Table 3). From these results, it can be seen that the expression of RDM1 is correlated with most types of macrophages, which also suggests that RDM1 may play a role in regulating the polarization of macrophages. The weak correlation of the expression of RDM1 with immune markers of dendritic cells (DCs) also suggests that *RDM1* gene expression is related to the penetration of DCs. Finally, most of the immune markers of T-cell exhaustion are significantly correlated with the expression of RDM1, suggesting that we need to pay attention to nonfunctional T cells. This finding also indicates that RDM1 may play a role in the immune escape of hepatocellular carcinoma.

**Table 3:** Correlation analysis between RDM1 and marker genes of immune cells in GEPIA

Description	Gene markers	LIHC				CHOL			
		Tumor		Normal		Tumor		Normal	
		R	*P*	R	*P*	R	*P*	R	*P*
B cell	CD19	0.23	1.10E-05	0.25	7.60E-02	−0.02	9.00E-01	0.09	8.10E-01
Monocyte	CD86	0.16	2.00E-03	0.06	7.00E-01	−0.30	7.60E-02	−0.51	1.60E-01
	CD115 (CSF1R)	0.05	3.50E-01	0.06	6.80E-01	−0.18	2.90E-01	−0.15	7.10E-01
TAM	CD68	0.09	9.00E-02	-0.02	8.90E-01	−0.09	5.80E-01	−0.55	1.30E-01
	IL10	0.06	2.90E-01	0.06	7.00E-01	0.18	3.00E-01	−0.24	5.40E-01
M1	IRF5	0.30	2.30E-09	0.06	6.90E-01	0.09	6.10E-01	−0.06	8.90E-01
Th1	STAT4	0.17	1.40E-03	0.14	3.20E-01	0.10	5.60E-01	0.26	5.10E-01
	STAT1	0.22	2.10E-05	0.18	2.10E-01	0.33	8.60E-01	−0.33	3.90E-01
	IFN-γ (IFNG)	0.19	1.70E-04	0.23	1.00E-01	0.06	7.30E-01	−0.08	8.30E-01
	TNF-ax (TNF)	0.12	2.50E-02	0.23	1.10E-01	−0.34	4.20E-02	−0.22	5.70E-01
Th2	STAT5A	0.15	5.20E-03	0.08	5.60E-01	−0.28	1.00E-01	−0.20	6.00E-01
T-cell exhaustion	PD-1 (PDCD1)	0.17	1.20E-03	0.32	2.40E-02	0.01	9.50E-01	−0.15	7.10E-01
	CTLA4	0.22	2.00E-05	0.47	6.10E-04	−0.07	9.50E-01	−0.39	2.90E-01
	LAG3	0.21	6.10E-05	0.20	1.70E-01	0.13	4.40E-01	−0.13	7.40-01
	TIM-3 (HAVCR2)	0.16	1.90E-03	−0.01	9.40E-01	−0.26	1.20E-01	−0.73	2.50E-02

### IHC of RDM1

**Figure 5:**
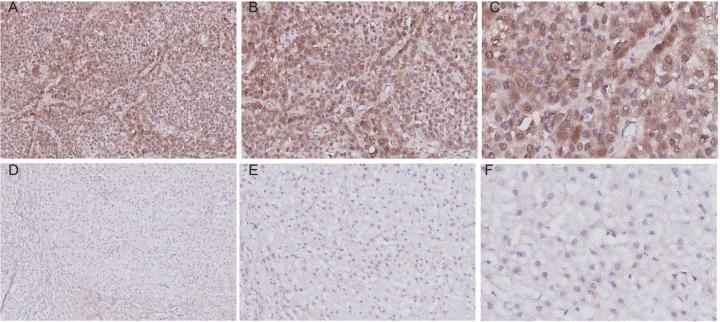
Representative images of immunostaining for RDM1 in LIHCand adjacent normal tissues (**A**) Immunostaining of LIHC tissues (×100). (**B**) Immunostaining of LIHC tissues (×200). (**C**) Immunostaining of LIHC tissues (×400). (**D**) Immunostaining of normal tissues (×100). (**E**) Immunostaining of normal tissues (×200). (**F**) Immunostaining of normal tissues (×400). The brown area in (A,B) marked the positive staining of RDM1 in adjacent normal tissues.

**Figure 6:**
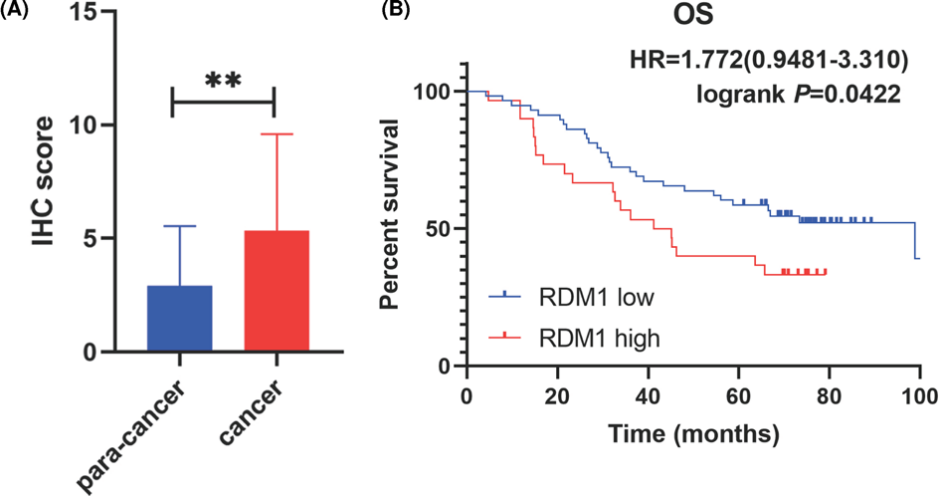
Statistical results of IHC were presented (**A**) Immunohistochemical scores of hepatocellular carcinoma and paracancerous tissues in 90 cases. (**B**) Relationship between RDM1 expression and prognosis and OS. **P<0.01.

Ninety pairs of LIHC and adjacent normal tissues were collected from the South Branch of the First People’s Hospital Affiliated to Shanghai Jiaotong University during the period of February 2014 to May 2019. The experiments were approved by the Ethical Committee of the First People’s Hospital Affiliated to Shanghai Jiaotong University and written informed consent was signed by each participant. Two pathologists unaware of the clinicopathological data assessed the immunostained slides independently. Analysis of RDM1 expression patterns in LIHC and paracarcinoma tissues was performed via Wilcoxon signed-rank tests in SPSS v.22.0. The median age of patients was 49.5 years (range: 21–73 years) and the majority (89%) were male. Of the total 90 patients, average survival of postoperative patients was 53.34 months (range: 4.2–108.23 months). Distant metastasis and local recurrence occurred in 13 (14%) and 11 (12%) patients, respectively. The immunostaining status of RDM1 protein expression in liver cancer tissues (Figure 5A–C) and the staining status in adjacent tissues (Figure 5D–F). The immunohistochemical staining score of liver cancer group was higher than that of adjacent cancer group (Figure 6A, *P*<0.01), RDM1. The expression is related to the OS of the patient (Figure 6B). However, the expression of RDM1 protein has no correlation with clinically relevant biochemical indicators and tumor size (Supplementary Figure S9).

## Discussion

RDM1 belongs to a family of proteins containing gene-binding motifs, and its sequence is similar to that of the DNA recombination and repair gene *RAD52* [15,40]. As cancer progresses, more mutations accumulate in the DNA repair proteins of the tumor [41]. Previous studies have shown that RDM1 potentiates RAD52–RAD51 signaling via p53-mediated transcriptional suppression [20,21]. p53 plays an important role in regulating cell growth and apoptosis by triggering cell growth arrest or apoptosis through the regulation of various downstream targets [42].

However, whether the regulation of RDM1 by p53 is the main mechanism leading to the decreased proliferation and enhanced apoptosis of RDM1 knockdown cells remains to be further investigated.

The biological targets and function of RDM1 are still unknown. Here, we found that high mRNA expression of RDM1 is related to the occurrence and development of cancer. The high expression of RDM1 is closely related to the prognosis of large logarithmic tumors, especially in patients with hepatocellular carcinoma, poorer OS and PFS, of which PFS is the most significant factor. In addition, our analysis showed that in hepatocellular carcinoma, the levels of immune infiltration and various immune markers are correlated with the expression level of RDM1. Our research provides insights to better understand the potential role of RDM1 in tumor immunology and its application as a new immune marker for LIHC.

In the present study, we used the Oncomine and TIMER databases to reveal significant differences between RDM1 expression in many different tumor types and normal tissues.

The Oncomine data showed that RDM1 expression is up-regulated in colon cancer, breast cancer, gastric cancer, lung cancer, melanoma, ovarian cancer and prostate cancer compared with normal tissue. The TCGA database showed that RDM1 expression is significantly correlated with most tumor types. The different expression changes of RDM1 across cancers may be related to the different database collection methods and different tumor biological mechanisms. At the same time, the prognostic value of RDM1 across cancers was well elucidated. In the Oncomine and PrognoScan databases, high RDM1 expression was associated with poor prognoses of ovarian, bladder, breast, brain, lung and liver cancer, ACC, KIRP and LGG. Even more remarkable, the prognostic survival rate of LIHC patients was affected by changes in RDM1 expression, which correlated to the following: stage (1+2, 2+3, 3+4), grade, AJCC_T, vascular invasion and hepatitis virus status. Among them, the expression of RDM1 and the prognosis of liver cancer increased with the increase in the LIHC stage and grade. An increased correlation significance corresponded to a higher HR ratio, indicating that RDM1 expression is significantly up-regulated in patients with advanced hepatocellular carcinoma. Advanced hepatocellular carcinoma corresponds to a more malignant degree of differentiation, atypia and cell structure in hepatocellular carcinoma patients, as well as a larger tumor size, which involves greater risks of vascular invasion and extrahepatic metastasis.

Patients with LIHC had a higher hazard risk ratio for vascular invasion, and vascular invasion is one of the mechanisms of LIHC metastasis. Additionally, this result reflects that high RDM1 expression is related to invasion and metastasis of hepatocellular carcinoma. It was also found that there was a more significant risk ratio in hepatitis-free patients with high RDM1 expression than in carriers, suggesting that increased RDM1 expression in hepatitis-free patients was associated with a poor prognosis. This finding also indicated that there may be a connection between hepatitis virus and RDM1, but further studies are needed to prove this phenomenon. Another key finding of the present study was that RDM1 is related to immunity to multiple cancer types. In particular, RDM1 is significantly related to the level of immune infiltration in LIHC. In LIHC, the expression of RDM1 is strongly positively correlated with tumor purity and infiltration of B cells, CD8^+^ T cells, macrophages and DCs. These findings indicate that RDM1 plays an important role in regulating tumor immunity and therefore affects the prognosis of LIHC. In CHOL, RDM1 showed a negative correlation with immune cell infiltration. The RDM1 expression levels were correlated with monocyte markers (CD86, CD115), TAM markers (CD68, IL-10), M1 macrophage marker (IRF5) and M2 macrophage markers (CD163, VSIG4, MS4A4A) in LIHC, suggesting that RDM1 can participate in macrophage polarization. Through further observation, it was found that the expression of RDM1 was significantly correlated with markers (CD3D, CD3E, CD2, STAT4, STAT1, IFN-disc, TNF-ax, FOXP3, CCR8, PD-1, CTLA4, LAG3, TIM-3c) of T cells and their subsets. For the Treg immune marker forkhead box protein 3 (FOXP3), in recent years, it has been proposed that overexpression of FOXP3 promotes the progression of LIHC, whereas FOXP3 knockdown inhibits tumor growth in LIHC mice [43,44]. Due to the high correlation of RDM1 expression and T-cell exhaustion, we suspect that T-cell immunity does not work in LIHC patients with high RDM1 expression. Previous studies have shown that the immune marker of T-cell exhaustion, PD-1 is a T-cell checkpoint and can inhibit the activation of T cells and regulate T-cell fatigue. Additionally, PD-1 and its ligand PD-L1 play an important role in helping tumor cells escape [45,46]. It has been shown that T cells with high expression of PD-1 are more exhausted than T cells without high expression of PD-1 in hepatocellular carcinoma cell [12]. This change can affect the antitumor function of T cells and lead to a poor prognosis in LIHC. However, this hypothesis needs further study. Through analysis of the above immune markers and immune checkpoints, we can clearly understand the profound impact of RDM1 expression on the occurrence and development of hepatocellular carcinoma, as well as its immune escape in the tumor microenvironment.

These results confirm the influence of RDM1 in hepatocellular carcinoma and highlight a possible molecular mechanism. Of course, follow-up experiments are required to validate these findings. In summary, our findings show that RDM1 plays an indispensable role in regulating the infiltration of immune cells in LIHC.

The results of the present study suggest that RDM1 plays a role in the regulation and recruitment of certain immune cells and their markers in LIHC. These data reveal the relationship between RDM1 expression and the survival of LIHC patients and the level of immune infiltration. The present study still has certain limitations. We used the Oncomine, TIMER, GEPIA, Kaplan–Meier plotter and PrognoScan databases and other published literature to obtain relevant data on the role of RDM1 in hepatocellular carcinoma but did not verify the results through basic experiments. Additionally, in some databases, the sample size was too small to be included in the statistics. An increased number of samples is needed to obtain more reliable data. As a result, the correlation between RDM1 and immune cell infiltration and clinical prognosis in hepatocellular carcinoma requires relevant basic experiments for validation.

In the present study, in order to verify the reliability of the results, the immunohistochemical staining count was selected to further confirm the prognostic value of RDM1. The results showed that RDM1 expression was up-regulated in liver cancer tissues, and the immunostaining score was higher than that of adjacent tissues. The increased expression of RDM1 predicts a poor prognosis. The correlation between survival was further confirmed, but no correlation with tumor size and number was found. Unfortunately, the immunohistochemical technique used in the present study has limitations in distinguishing pure cytoplasmic expression from the combination of cytoplasmic and nuclear expression.

## Conclusion

In conclusion, the present study jointly explores the role of *RDM1* gene expression in hepatocellular carcinoma from the two aspects of immune cell infiltration and prognostic survival. The results of the present study suggest that RDM1 is potentially a novel prognostic marker for different tumor types that can assess the level of immune cell infiltration, especially in hepatocellular carcinoma. High expression of RDM1 promote a poor prognosis. In summary, these findings strongly suggest that RDM1 is a valuable prognostic marker for hepatocellular carcinoma.

## Supplementary Material

Supplementary Figures S1-S9 and Table S1

## Data Availability

The data that support the findings of the present study are available in TCGA at [https://portal.gdc.cancer.gov] and Gene Expression Omnibus (GEO) at [https://www.ncbi.nlm.nih.gov/gds/]; these databases are public databases.

## Abbreviations

ACC: adrenocortical carcinoma
AJCC_T: American Joint Committee on Cancer TNM system-T tumor size
CHOL: cholangiocarcinoma
CI: Confidence interval
DC: dendritic cell
GEPIA: Gene Expression Analysis Interactive Analysis
GTEx: Genotype-Tissue Expression
HR: HazardRatio
IHC: immunohistochemistry
KIRP: kidney renal papillary cell carcinoma
LGG: lower grade glioma
LIHC: liver hepatocellular carcinoma
mRNA: messenger RNA
NK: natural killer cell
OS: overall survival
PD-1: programmed cell death protein-1
PD-L1: programmed death receptor ligand-1
PFS: progression-free survival
RDM1: RAD52 motif-containing protein 1
TAM: tumor-associated macrophage
TCGA: The Cancer Genome Atlas
TIMER: Tumor Immune Assessment Resource
TNM: tumor node metastasis classification
Treg: regulatory T cell
