# Supplementary material for: Correlation analysis of RDM1 gene with immune infiltration and clinical prognosis of hepatocellular carcinoma
Source: Biosci Rep. 2021 Sep 17;41(9):BSR20203978. doi: 10.1042/BSR20203978 (PMC8450314; doi:10.1042/BSR20203978)
Supplement: Supplementary Figures S1-S9 and Table S1 [file BSR-2020-3978_supp.pdf]

Supplement figure 1: The relationship between RDM1 expression and the prognosis of various cancers. (A):Overall survival and disease-free survival of ACC and BLCA. (B): Overall survival and disease-free survival of BRCA and CESC. (C):Overall survival and disease-free survival of CHOL and COAD. (D):Overall survival and disease-free survival of DLBC and ESCA. (E):Overall survival and disease-free survival of GBM and HNSC.

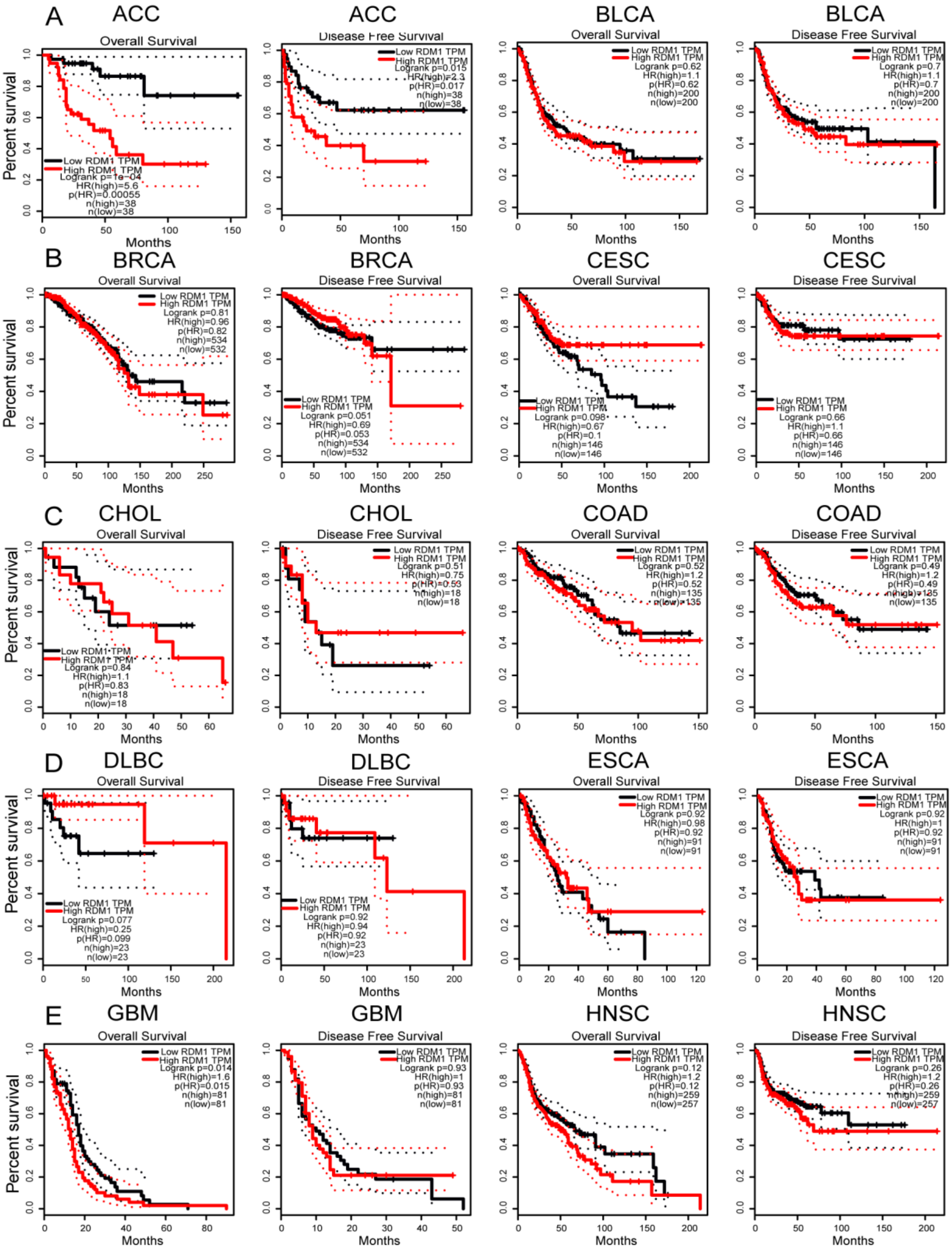

Supplement figure 2: The relationship between RDM1 expression and the prognosis of various cancers. (F):Overall survival and disease-free survival of KICH and KIRC. (G): Overall survival and disease-free survival of KIRP and LAML. (H):Overall survival and disease-free survival of LGG and LIHC. (I):Overall survival and disease-free survival of LUAD and LUSC. (J):Overall survival and disease-free survival of MESO and OV.

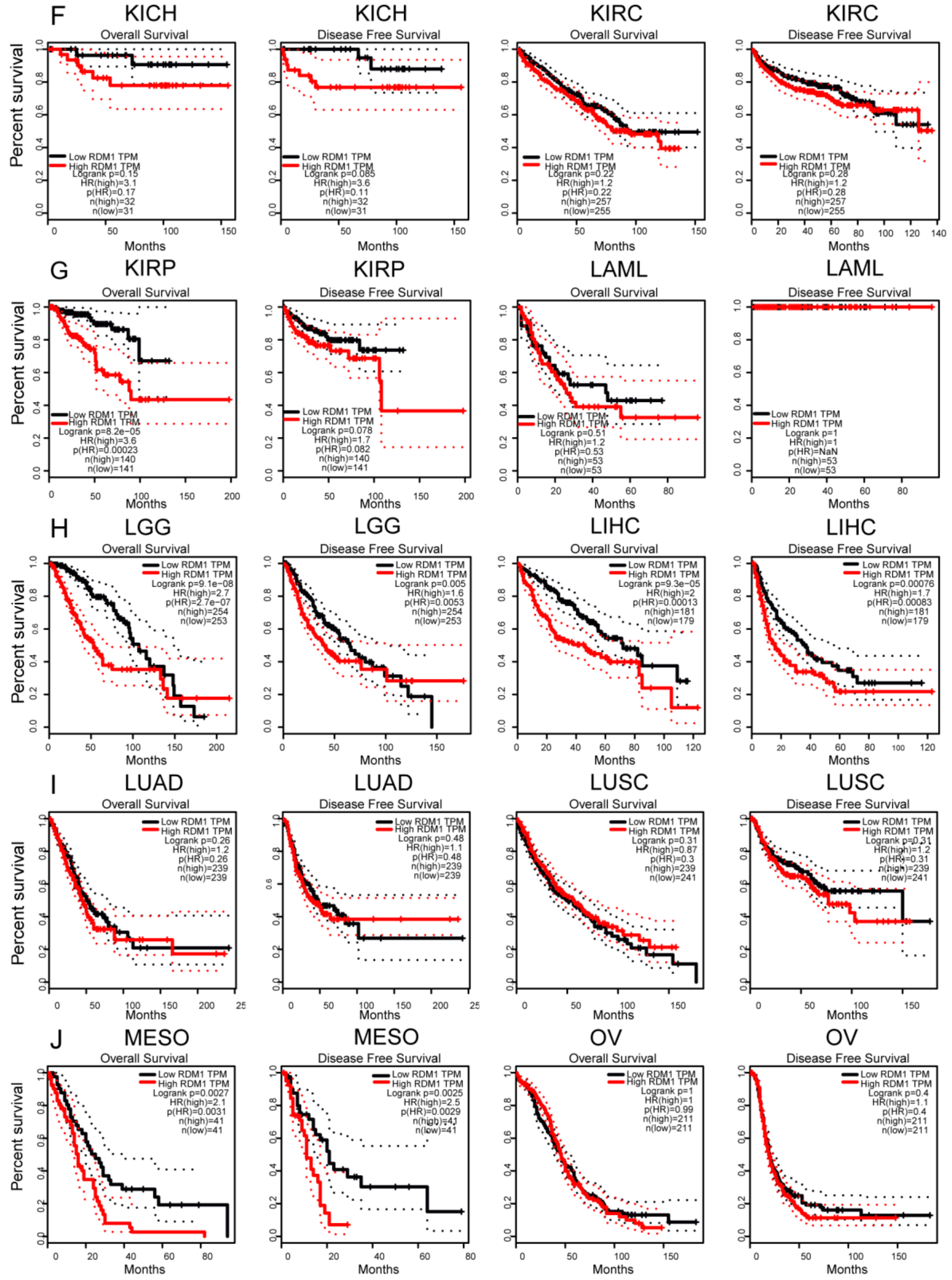

Supplement figure 3: The relationship between RDM1 expression and the prognosis of various cancers. (K):Overall survival and disease-free survival of PAAD and PCPG. (L): Overall survival and disease-free survival of PRAD and READ. (M):Overall survival and disease-free survival of SARC and SKCM. (N):Overall survival and disease-free survival of STAD and TGCT. (O):Overall survival and disease-free survival of THCA and THYM

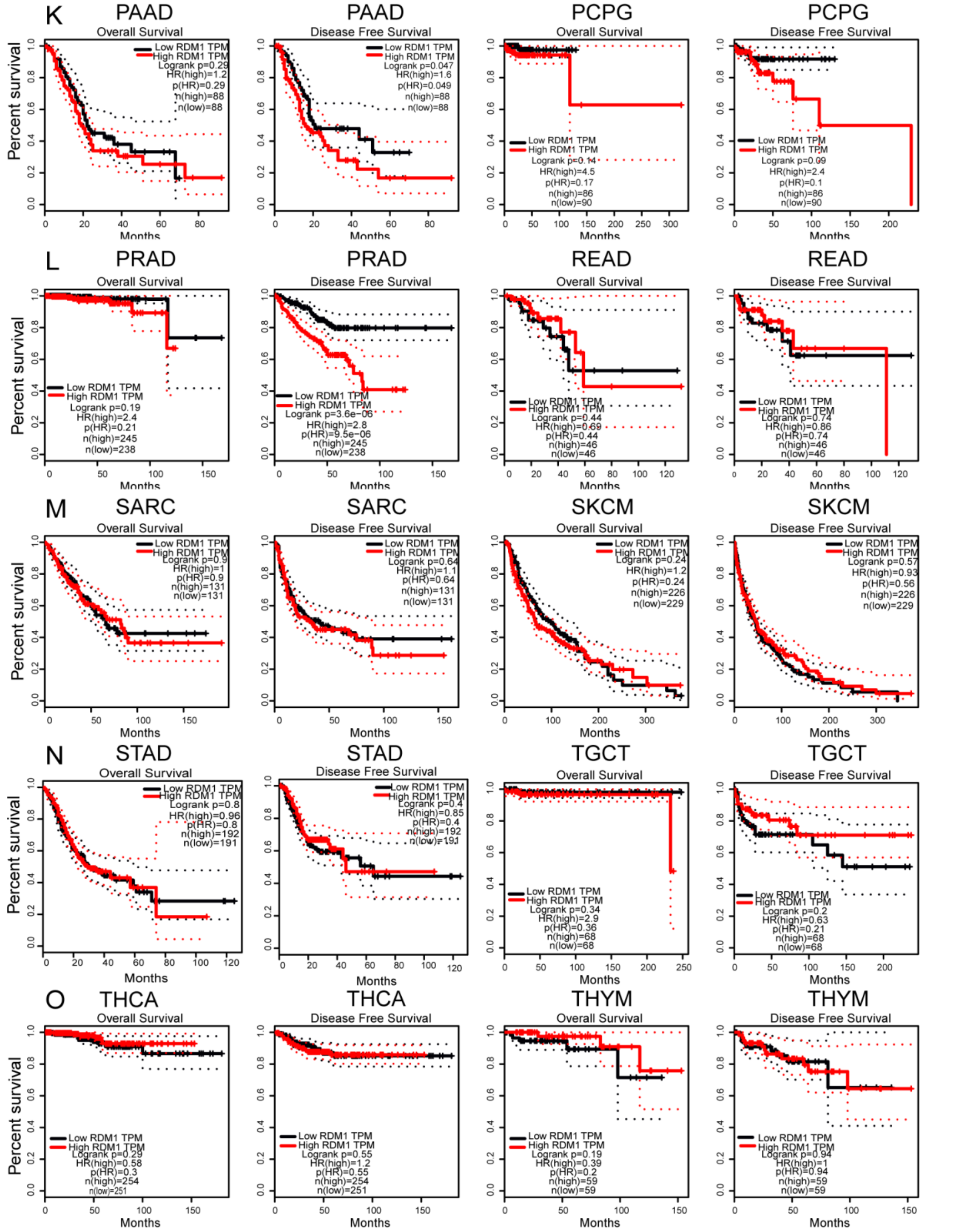

Supplement figure4: The relationship between RDM1 expression and the prognosis of various cancers.
(P):Overall survival and disease-free survival of UCEC and Overall survival of UCS. (Q): disease-free survival of UCS and Overall survival and disease-free survival of UVM.

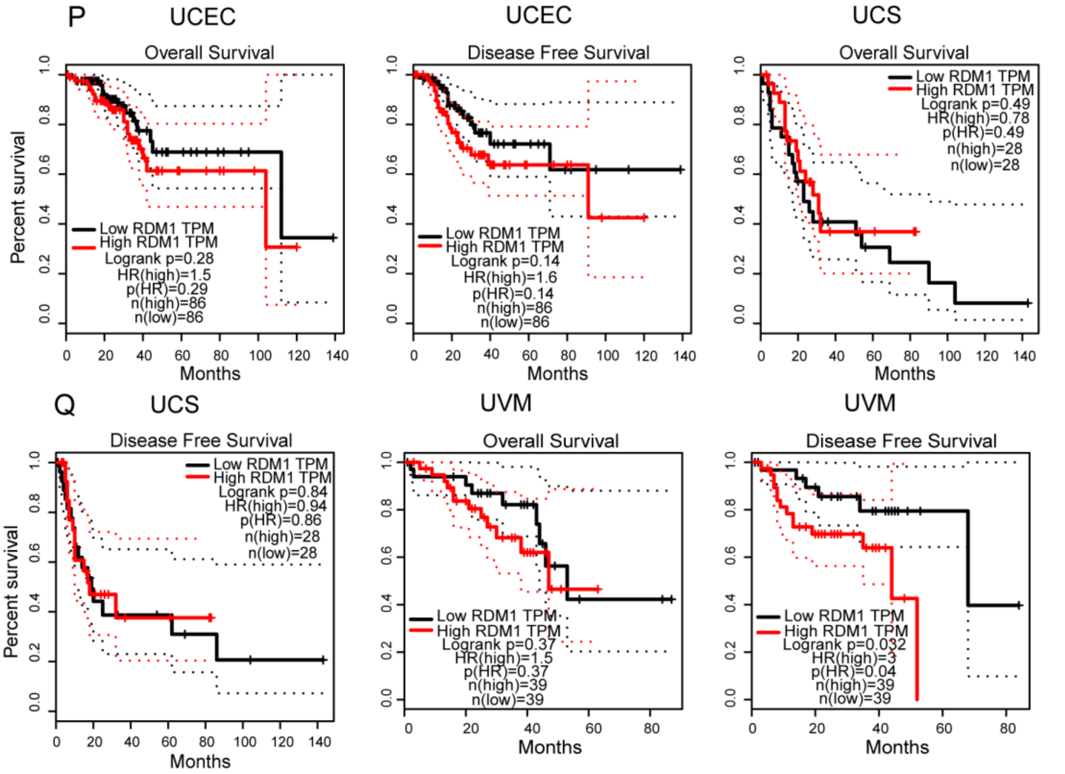

Supplement figure5:The relationship between RDM1 expression and immune cell infiltration.(A):Adrenocortical carcinoma.(B):Bladder Urothelial Carcinoma.(C):Breast invasive carcinoma.(D):Breast invasive carcinoma basal-like.(E):Breast carcinoma with HER -2 overexpression.(F): Breast carcinoma with type Luminal.(G):Cervical squamous cell carcinoma and endocervical.(H):Colon adenocarcinoma.

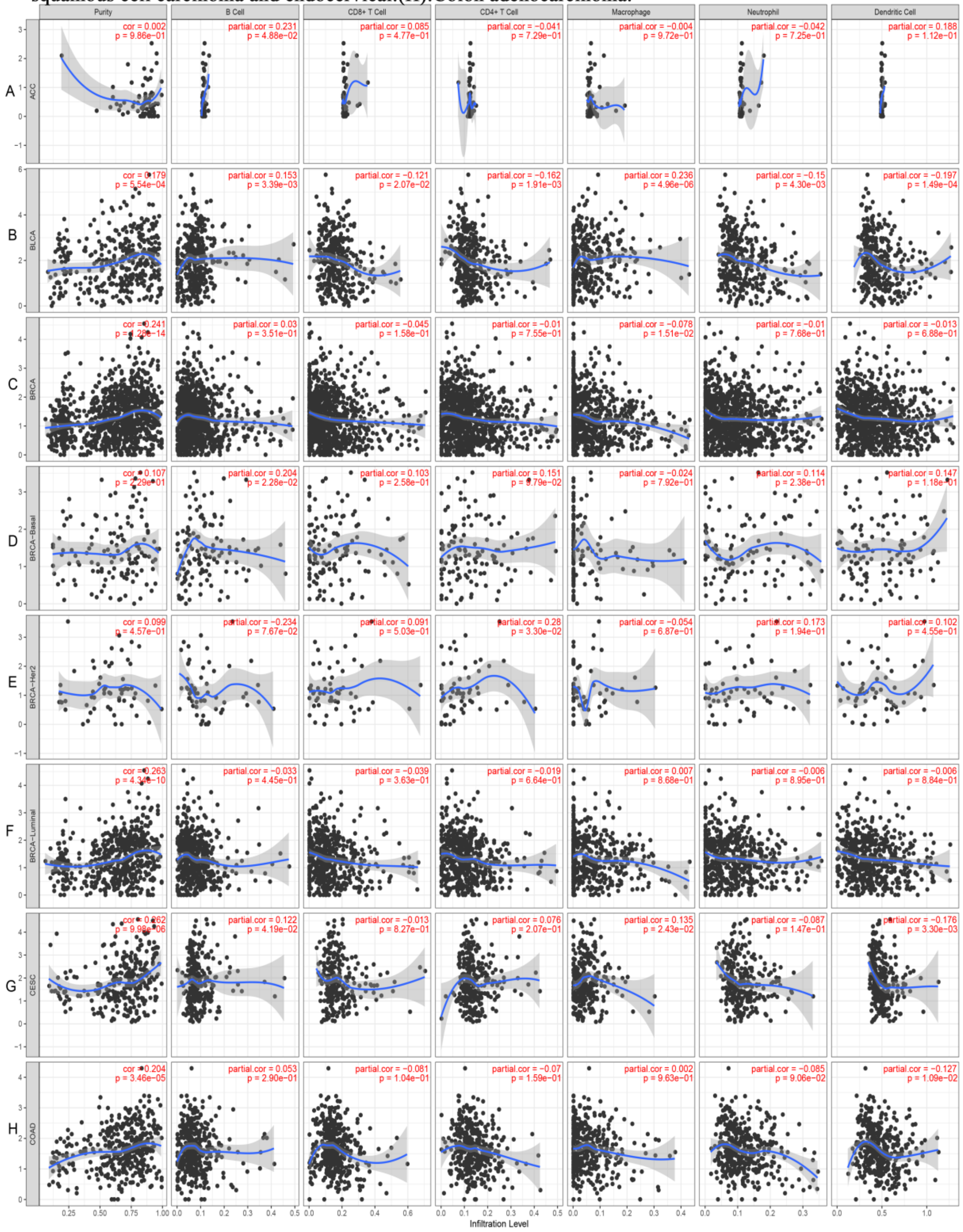

Supplement figure6:The relationship between RDM1 expression and immune cell infiltration.(I): Lymphoid Neoplasm Diffuse Large B-cell Lymphoma.(J):Esophageal carcinoma.(K):Glioblastoma multiforme.(L):Head and Neck squamous cell carcinoma.(M):Head and Neck squamous cell carcinoma with HPV possitive.(N): Head and Neck squamous cell carcinoma with HPV negative(O):Kidney Chromophobe.(P):Kidney renal clear cell carcinoma.

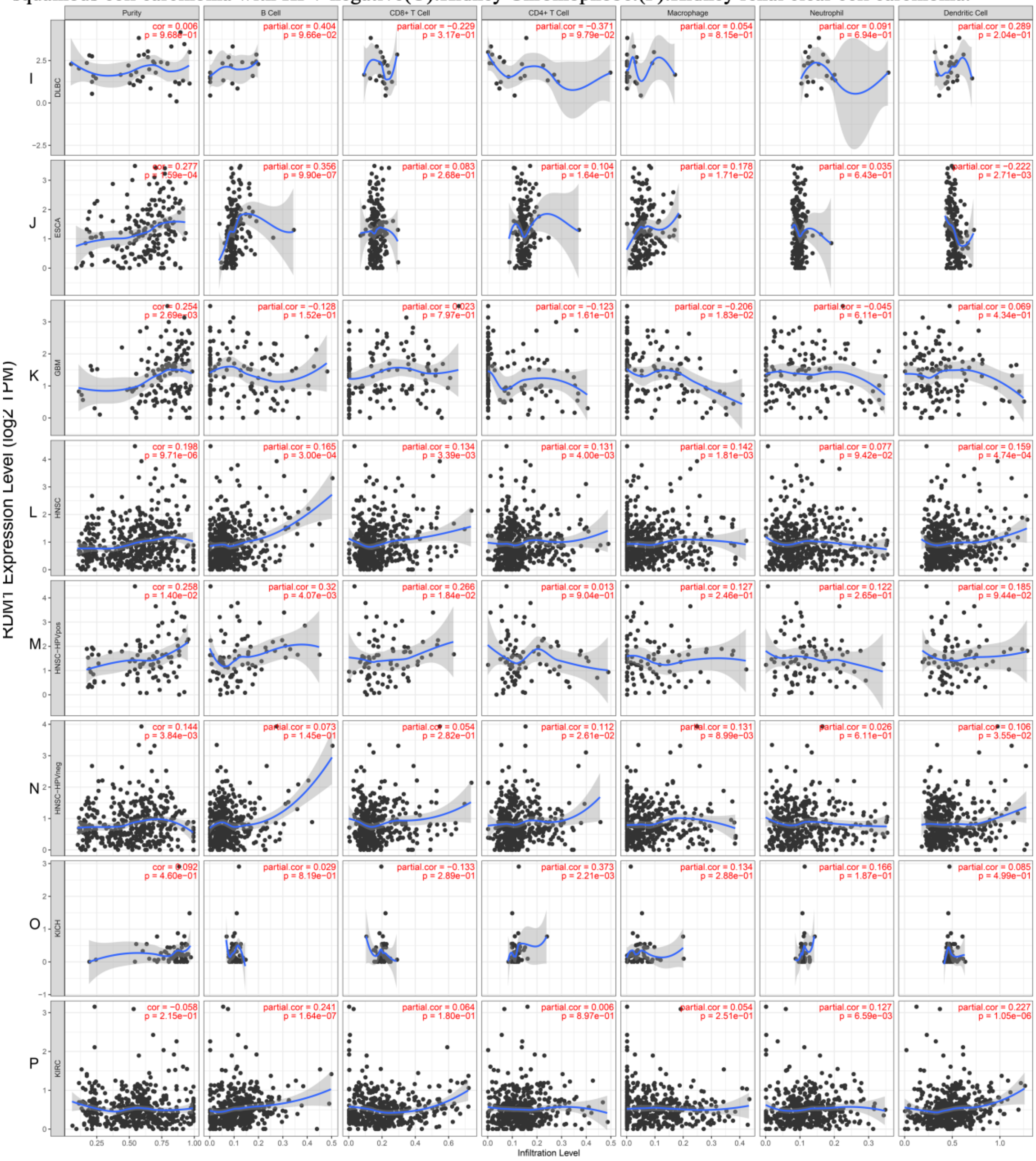

Supplement figure7:The relationship between RDM1 expression and immune cell infiltration.(Q):Kidney renal papillary cell carcinoma.(R):Brain Lower Grade Glioma.(S):Lung adenocarcinoma.(T): Lung squamous cell carcinoma.(U):Mesothelioma.(V): Ovarian serous cystadenocarcinoma(W):Pancreatic adenocarcinoma.(X):Pheochromocytoma and Paraganglioma.

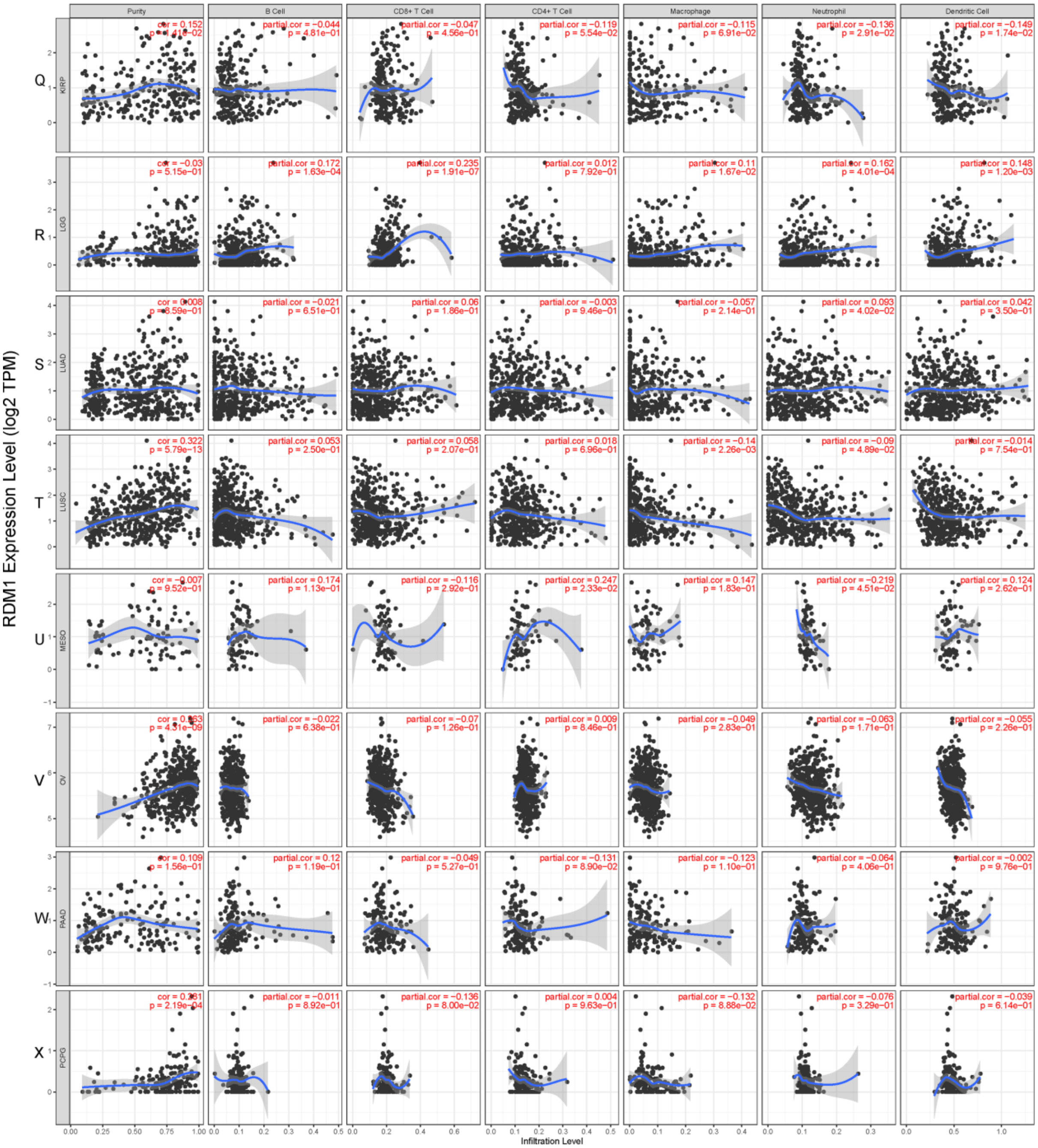

:

Supplement figure8:The relationship between RDM1 expression and immune cell infiltration.(Y):Kidney renal papillary cell carcinoma.(Z):Brain Lower Grade Glioma.(AA):Lung adenocarcinoma.(AB): Lung squamous cell carcinoma.(AC):Mesothelioma.(AD): Ovarian serous cystadenocarcinoma(AE):Pancreatic adenocarcinoma.(AF):Pheochromocytoma and Paraganglioma.

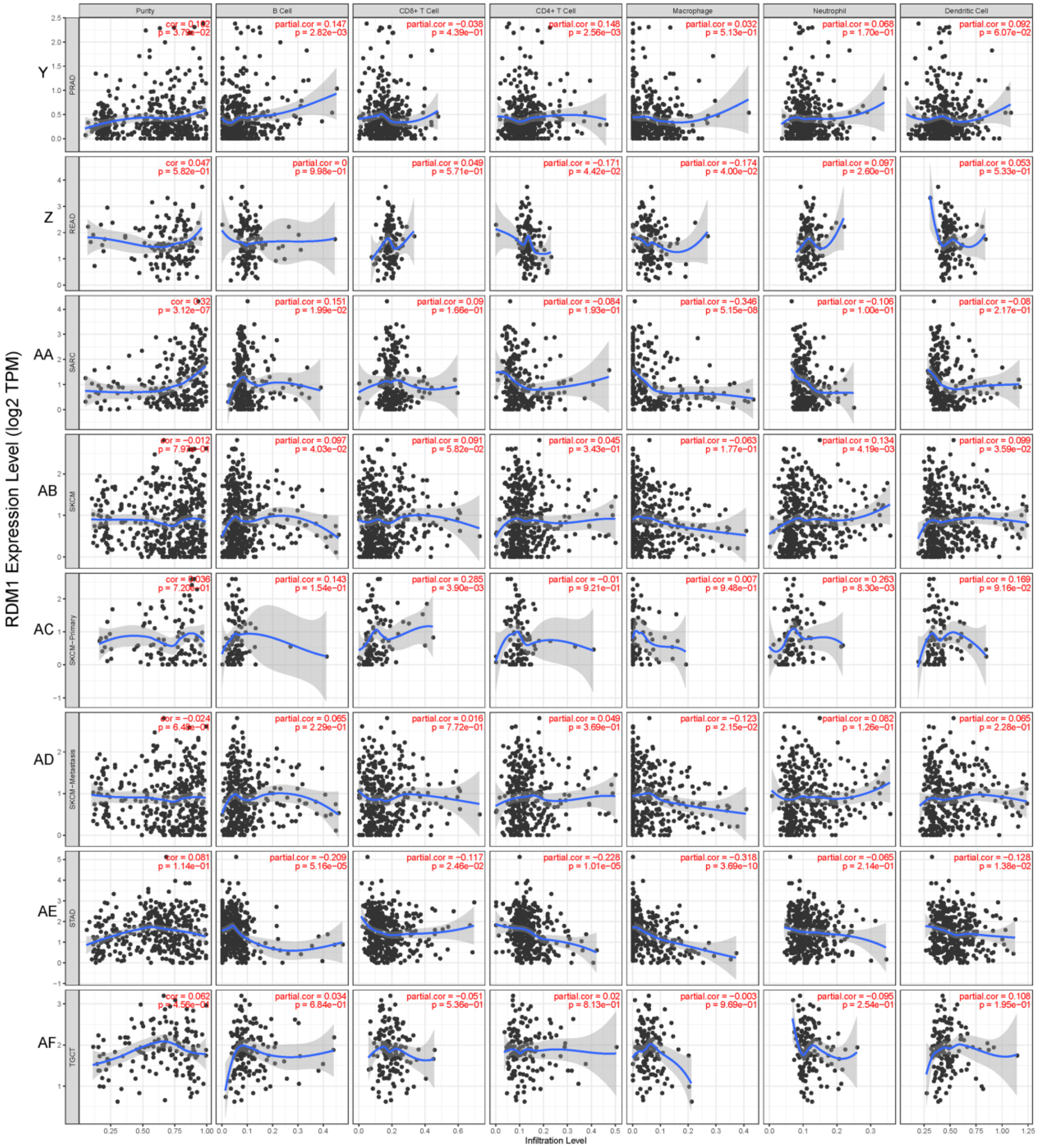

Supplement figure 9:Relationship between RDM1 expression and clinical biochemical indexes, tumor size and number in 90 patients with hepatocellular carcinoma .(A):tumor size .(B):number of tumor. (C):degree of differentiation (D):alanine amiotransferase(ALT).(E): direct bilirubin(DB) .(F) total bilirubin(TB). (G): albumin(ALB).(H):γ-glutamyl transpeptadase(GGT).(I):Alpha fetoprotein(AFP).

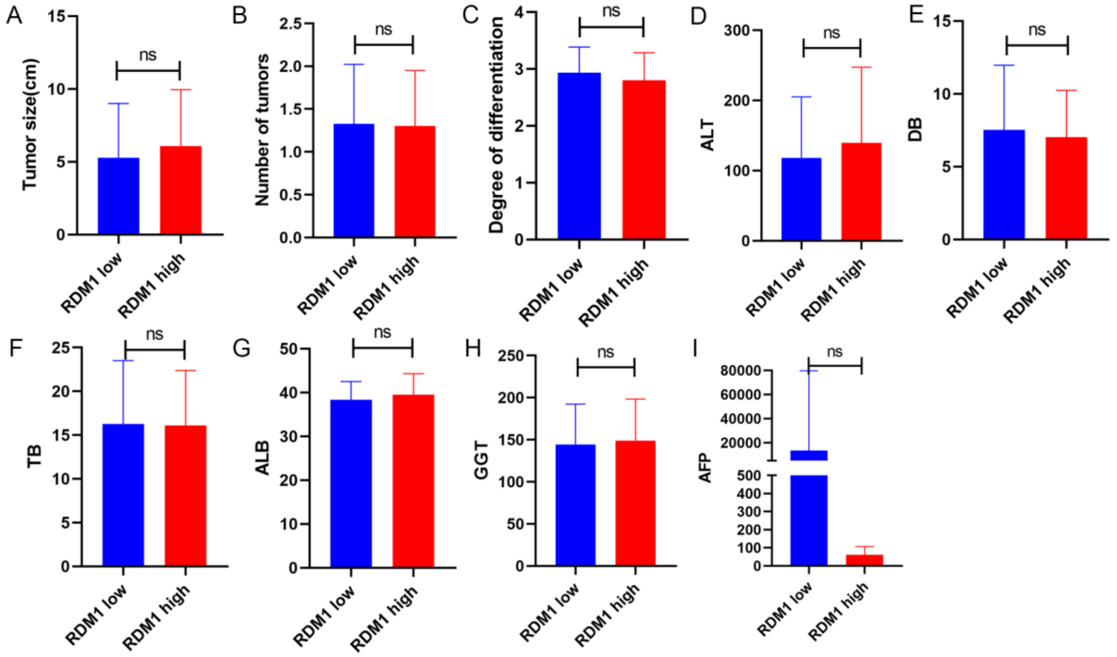

Supplementary Table .1 RMD1 mRNA expression levels of different types of tumors were found in the Oncomine database

Cancer	Cancer type	*P*-value	Fold change	Rank (%)	Sample	Reference (PMID)
Brain and CNS	Glioblastoma	1.13E-06	-6.492	10%	22	16697959
Breast	Mucinous Breast Carcinoma	1.00E-03	4.603	4%	4	TCGA
	Invasive Breast Carcinoma	1.65E-13	2.738	8%	76	TCGA
	Invasive Ductal Breast Carcinoma	1.69E-18	2.705	10%	389	TCGA
	Invasive Lobular Breast Carcinoma	4.85E-08	2.325	10%	36	TCGA
	Intraductal Cribriform Breast Adenocarcinoma	2.00E-02	3.116	16%	3	TCGA
	Mixed Lobular and Ductal Breast Carcinoma	4.30E-02	1.705	31%	7	TCGA
	Invasive Ductal Breast Carcinoma	4.70E-02	1.583	11%	5	17389037
Colorectal	Colon Carcinoma	8.79E-06	2.083	13%	5	20957034
	Colon Carcinoma Epithelia	6.48E-04	1.571	19%	5	20957034
	Rectal Adenoma	2.01E-04	2.186	13%	7	18171984
	Colon Adenoma	1.74E-07	2.285	15%	25	18171984
	Colorectal Carcinoma	1.80E-02	1.711	36%	70	20143136
Gastric	Gastric Intestinal Type Adenocarcinoma	1.66E-04	2.379	23%	26	19081245
	Gastric Mixed Adenocarcinoma	2.80E-02	2.05	29%	4	19081245
Kidney	Renal Oncocytoma	1.00E-03	-8.618	3%	4	19445733
	Chromophobe Renal Cell Carcinoma	2.30E-02	-3.171	11%	4	19445733
	Renal Wilms Tumor	1.00E-02	4.792	11%	4	19445733
Lung	Large Cell Lung Carcinoma	6.73E-05	1.51	11%	19	20421987
	Lung Adenocarcinoma	1.16E-06	3.829	16%	226	22080568
Melanoma	Cutaneous Melanoma	4.60E-02	1.669	20%	14	18442402
other	Teratoma, NOS	4.75E-07	-1.797	10%	14	16424014
	Seminoma, NOS	8.40E-06	-1.584	13%	12	16424014
	Embryonal Carcinoma, NOS	2.42E-05	-1.641	17%	15	16424014
	Mixed Germ Cell Tumor, NOS	3.83E-05	-1.525	20%	41	16424014
	Testicular Seminoma	1.10E-02	-1.582	17%	3	15994931
Ovarian	Ovarian Serous Adenocarcinoma	1.22E-06	9.668	4%	43	19486012
Prostate	Prostate Carcinoma	4.45E-04	1.563	10%	58	22722839
